# Clustering analysis of proteins from microbial genomes at multiple levels of resolution

**DOI:** 10.1186/s12859-016-1112-8

**Published:** 2016-08-31

**Authors:** Leonid Zaslavsky, Stacy Ciufo, Boris Fedorov, Tatiana Tatusova

**Affiliations:** National Center for Biotechnology Information, National Library of Medicine, National Institutes of Health, Bethesda, 20894 MD USA

**Keywords:** Protein, Cluster, Clustering, Microbial, Procaryotic, Core-periphery, Multiscale, Multiresolution, Knowledge discovery, Data mining, Parallel processing, Parallel computing

## Abstract

**Background:**

Microbial genomes at the National Center for Biotechnology Information (NCBI) represent a large collection of more than 35,000 assemblies. There are several complexities associated with the data: a great variation in sampling density since human pathogens are densely sampled while other bacteria are less represented; different protein families occur in annotations with different frequencies; and the quality of genome annotation varies greatly. In order to extract useful information from these sophisticated data, the analysis needs to be performed at multiple levels of phylogenomic resolution and protein similarity, with an adequate sampling strategy.

**Results:**

Protein clustering is used to construct meaningful and stable groups of similar proteins to be used for analysis and functional annotation. Our approach is to create protein clusters at three levels. First, tight clusters in groups of closely-related genomes (species-level clades) are constructed using a combined approach that takes into account both sequence similarity and genome context. Second, clustroids of conservative in-clade clusters are organized into *seed* global clusters. Finally, global protein clusters are built around the the *seed* clusters. We propose filtering strategies that allow limiting the protein set included in global clustering.

The in-clade clustering procedure, subsequent selection of clustroids and organization into *seed* global clusters provides a robust representation and high rate of compression. Seed protein clusters are further extended by adding related proteins. Extended seed clusters include a significant part of the data and represent all major known cell machinery. The remaining part, coming from either non-conservative (unique) or rapidly evolving proteins, from rare genomes, or resulting from low-quality annotation, does not group together well. Processing these proteins requires significant computational resources and results in a large number of questionable clusters.

**Conclusion:**

The developed filtering strategies allow to identify and exclude such peripheral proteins limiting the protein dataset in global clustering. Overall, the proposed methodology allows the relevant data at different levels of details to be obtained and data redundancy eliminated while keeping biologically interesting variations.

**Electronic supplementary material:**

The online version of this article (doi:10.1186/s12859-016-1112-8) contains supplementary material, which is available to authorized users.

## Background

Microbial genomes at the National Center for Biotechnology Information (NCBI) represent a large collection of more than 35,000 assemblies from more than 5,000 species, with almost 40M unique proteins [1, 2]. Protein clustering is used to construct meaningful and stable groups of similar proteins to be analyzed and annotated, and serve as targets for efficient searching. There are several complexities associated with the data: the genomes in the dataset have different levels of sequence and assembly quality and large variation in sampling density; certain sets of related genomes, usually human pathogens, are densely sampled while other bacteria are less represented and sometimes sampled very coarsely (genomic and proteomic structure of a densely-sampled group of related strains is usually described by the concept of pan-genome [3–9]). Another factor contributing to the complexity of the analysis is a large variation in frequencies with which proteins from different families appear in genomes: “core proteins” occur at one end of the spectrum, unique proteins at another end, and “accessory proteins” in between (with some proteins partial in draft assemblies). In order to extract useful information from these complex data, the analysis needs to be performed at multiple levels of phylogenomic resolution and protein similarity, and an adequate sampling strategies.

Protein clusters are groups of similar (homologous) proteins that most likely share the same or similar function. Clustering procedure must possess a certain degree of stability and robustness and allow compression of information in comparison to the non-clustered representation. It is desirable that clusters consist of orthologs (protein coding regions that evolved from a common ancestral gene by speciation), while paralogs (genes related by duplication within a genome) stay in different clusters [10]. However, the ortholog-paralog distinction does not completely reflect the complexity of group relationships of homologous genes [11]. We make an effort to separate paralogs at the level of species-level genome groups (clades) using genomic context [12–18]. Since most microbial genomes at NCBI are draft genomes, local genomic context is utilized [19]. At the global level, we do not make a distinction between orthologous and paralogous proteins.

Here we present an efficient approach utilizing hierarchical clustering at several resolution levels. While large-scale hierarchical protein clustering is well-described in the literature [20–22], and methods for redundancy-elimination have been described by several authors [23–25], brute-force hierarchical clustering, even with a step of redundancy-elimination, becomes more expensive and less robust with the growth in the amount and complexity of data.

We construct protein clusters at three levels. First, in-clade protein clusters - tight protein clusters in groups of closely-related genomes (clades) are built. Then representaive proteins (*clustroids*) of conservative in-clade clusters are organized into *seed* global clusters. Clustroids of inclade clusters were selected as protein sequences providing minimal weighted average distance to other protein sequences in the clusters, where weight of each protein sequence was a number of coding regions in non-clonal genomes in the cluster encoding it. Finally, global protein clusters are built around the *seed* clusters. In-clade clustering with subsequent selection of clustroids and organizing them into *seed* global clusters provides a robust representation and high rate of compression in extended *seed* clusters. However, the proteins that are outside of the extended *seed* clustering set do not group together well. Processing of these proteins requires significant computational resources and results in a large number of questionable clusters. Such a pervasive behavior known as the *core-periphery* problem has been observed in many other areas of network analysis [26–28] where *peripheral* objects behaved very different from ones with high degree of centrality. We propose filtering strategies that allow limiting the protein set included in global clustering.

## Methods

Microbial genomes with full and nearly-full genome representation and good quality are organized in groups of closely-related genomes (species-level clades) constructed using ribosomal protein markers [1, 29, 30], Non-redundant representative genomes are selected in the groups of near-clonal genomes in each clade using the complete-linkage hierarchical clustering algorithm based on pairwise genomic BLAST with 95 % identity cut-off (there is the following order of preferences in selection of a representative genome: (1) clade (species) reference or representative; (2) included in KEGG database; (3) an annotated genome).

We extended our basic clustering procedure described in [31]. The similarity of proteins is determined from the aggregated BLAST hits obtained by BLASTp [32, 33] with e-value 10^−3^. The sequences are considered related if the minimum coverage and minimum similarity conditions are satisfied. We required at least 80 % similarity with 85 % coverage in in-clade clustering and at least 50 % similarity with 70 % coverage in all global clustering steps.

**Fig. 1:**
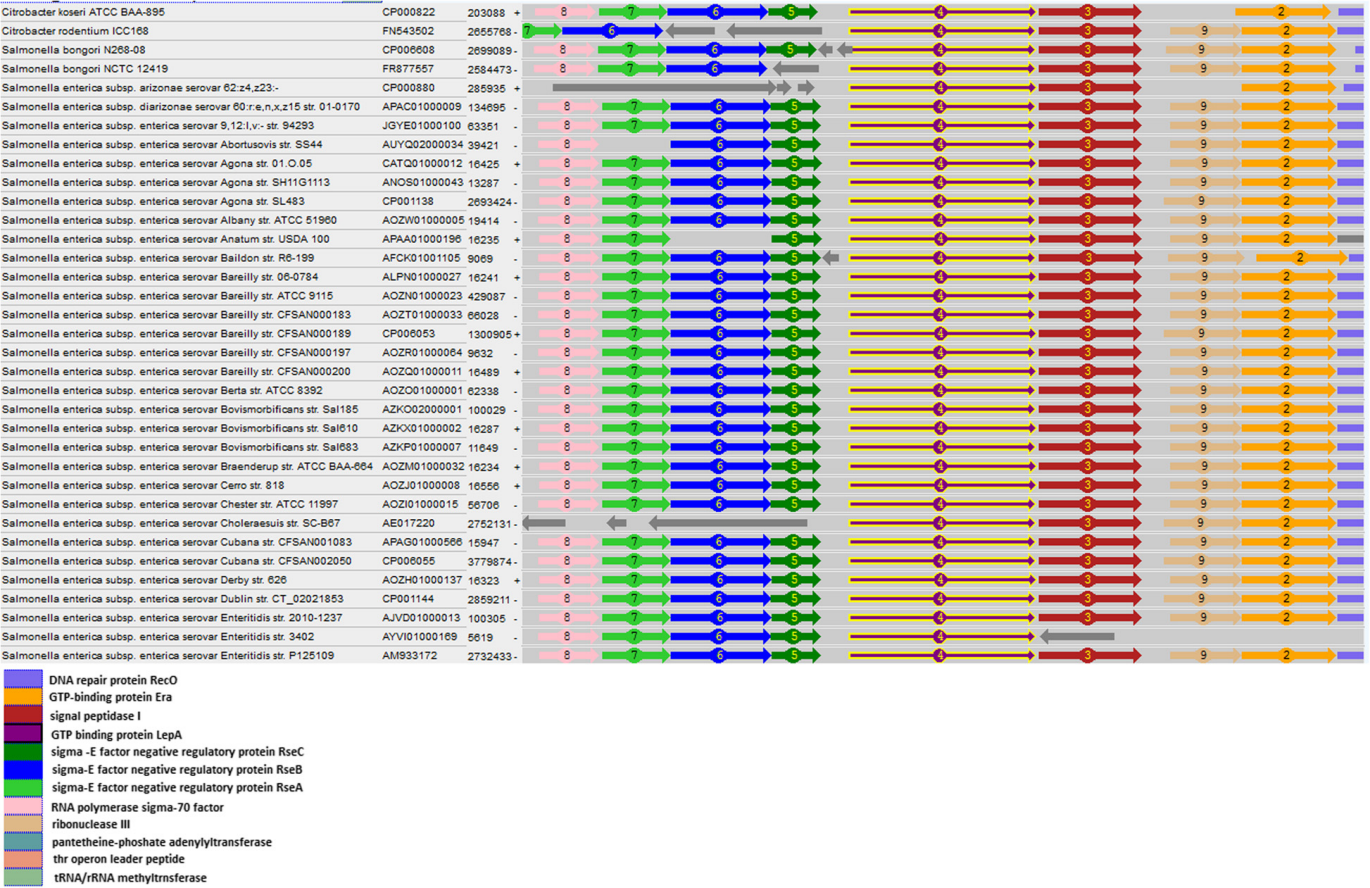
Local genomic neighborhood of the protein cluster containing the GTP-binding protein LepA (elongation factor) in Salmonella

In-clade clusters are constructed using a combined approach that takes into account both sequence similarity and local genome context [19]. First, sequence similarity clusters are calculated. Then, the genomic neighborhoods of proteins in each sequence-similarity cluster are analyzed using a moving window of 5-protein-length. Consequently, sub-clusters providing at least 3 out of 5 protein-similarity-cluster matches are selected (a protein map of local genomic neighborhood of the protein cluster containing the GTP-binding protein LepA (elongation factor) in Salmonella is shown in Fig. 1). Representaive proteins of inclade clusters (*clustroids*) were selected as protein sequences providing minimal weighted average distance to other protein sequences in the clusters, where weight of each protein sequence was a number of coding regions in non-clonal genomes in the cluster encoding it.

Two algorithms were considered for building global clusters around the *seed* clusters. The modified hierarchical clustering algorithm utilized our basic procedure with the following modification: when two sub-clusters, one containing *seed* proteins and another one not, are merged, the latter is not used when new distances are determined. The second procedure allowed extension of the *seed* clusters by adding non-seed proteins to the nearest *seed* cluster if they are compatible with *seed* clustroids there.

UCLUST and USEARCH [25] were used at different proceeding stages for redundancy elimination. In all cases we use values *wordlength 16, slots 400000009, maxrejects 64, maxaccepts 8*. The coverage and identity thresholds are selected differently for different steps: (1) Representatives from groups of near-identical sequences are selected before in-clade clustering is performed using coverage 100 % with identity 98 %; (2) Tight groups of proteins are formed for global clustering using coverage 85 % and identity 80 % approximately corresponding to parameters used in in-clade clustering. (3) Filtering which allows to find distant neighbors of the *seed* proteins, is performed using coverage 70 % with identity as low as 10 % (The built-in limitations of USEARCH prevent it from obtaining overly weak hits even if the the identity threshold is not set or set too low).

Many processing steps, such as computing BLAST hits, are naturally parallel. However, parallelization of clustering algorithms is a challenging problem which has attracted attention of computer scientists for years [20–22, 34–39]. While the single-linkage clustering algorithm can be run in parallel on a variety of architectures, other clustering algorithms require intensive communication between parallel processes. An alternative to an intensive exchange of data between the parallel processes is an iterative approach with an exchange of data between iterations [37]. However, in some cases, it is possible to partition data using a single-linkage-type algorithm and then concurrently perform clustering in each partition using a serial algorithm. Although the latter approach naturally produces a workload which is imbalanced to a certain degree, it does not require communication between the processes and is well-suited for large weakly-coupled distributed computer systems [40] as long as the load imbalance is tolerable. The hardware available at NCBI (a UGE Grid-Engine-based computer farm [41] and PanFS scalable storage system [42] connected through a powerful router), requires coarse-grained parallelization.

In our case, dataset reductions through selection of representative genomes in near-clonal groups and representative proteins in clade-level protein clusters allow to use the latter simplified approach, with differences in the partition sizes and resulting load balance to be acceptable. Our parallel clustering procedure is performed in three stages, each allowing concurrent processing: (1) The dataset is partitioned in disjoint sets using a parallel implementation based on a disjoint-set forest with union-by-rank heuristics [43, 44]; (2) Data are redistributed according to the partitioning; (3) Clustering is performed in each partition.

## Results

**Fig. 2:**
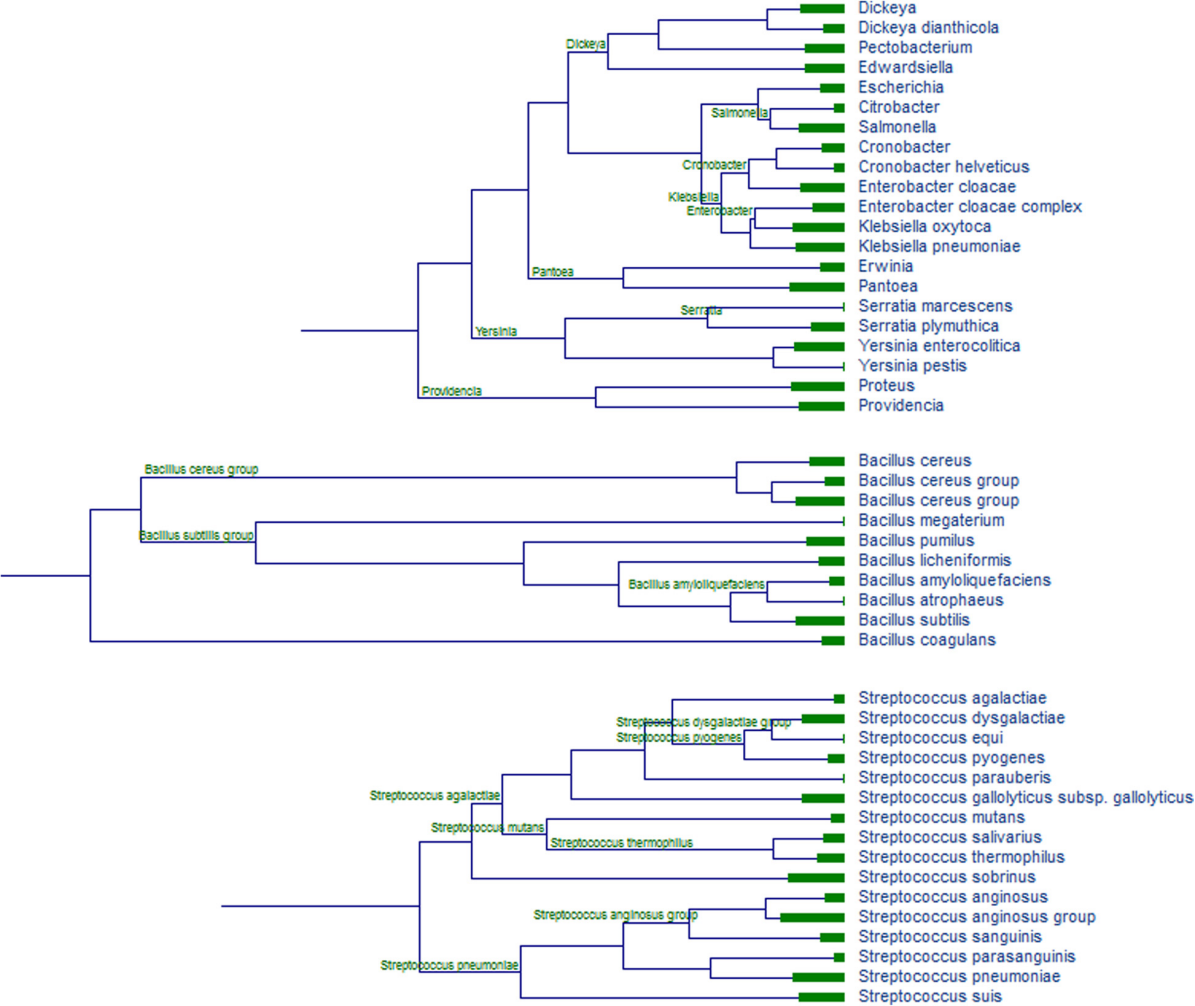
Parts of the clade tree around Salmonella, Bacillus and Streptococcus

Since NCBI production databases are updated in real time, the clustering analysis was performed on a snapshot created in November 2014. Prior to protein clustering, the groups of closely-related genomes (species-level clades) were constructed using ribosomal protein markers [1, 30] (Fig. 2 shows parts of the NCBI clade tree around *Salmonella*,*Bacillus* and *Streptococcus*). Within each clade, genomes are organized in tight (near-clonal) groups calculated using whole-genome BLAST alignment, and a non-redundant representative is selected in each tight genome group (see *Methods*). Table 1 shows the statistics for the most abundant clades (the statistics for all 131 abundant clades is shown in Additional file 1: Table S1).

**Table 1:** Statistics for the most abundant clades. The information for all 131 abundant clades is provided in Additional file 1: Table S1

Clade	Taxonomic content	No. annotated	No. nonclonal	No. protein	No. protein	No. conservative
Id		genomes	annotated genomes	coding regions	sequences	inclade clusters
19668	Escherichia, Shigella	2277	929	3303114	310023	3894
19507	Acinetobacter	749	280	774670	133653	3034
19252	Helicobacter pylori	309	216	254806	191419	1244
20139	Enterococcus genus	242	155	306721	33249	2106
20104	Streptococcus genus	347	139	163066	61589	1394
20137	Enterococcus genus	300	139	309061	45809	2314
19669	Salmonella, Citrobacter	638	134	478093	112833	3940
19672	Enterobacter, Escherichia, Klebsiella	350	132	593750	84168	4726
19537	Pseudomonas	229	118	622138	100992	5511
21194	Vibrio	271	118	433416	150390	4015
19400	Neisseria genus	204	109	162808	29688	1596
19988	Staphylococcus aureus	3827	108	235562	43260	2309
20122	Streptococcus agalactiae	285	103	165898	17943	1704
19671	Enterobacter Lelliottia	80	70	229896	102783	3476
20021	Bacillus	101	70	250224	101171	3919
20103	Streptococcus suis	92	69	97200	48055	1541
19543	Pseudomonas	108	68	219354	114229	3551
19270	Campylobacter jejuni	97	63	85618	29112	1444
20116	Streptococcus mutans	165	62	100740	28671	1672
19993	Staphylococcus genus	92	59	114655	23197	2014

The dataset contains 23,491 annotated assemblies, with 11,012 of them selected as representatives in near-clonal groups. The representative assemblies contain 40,362,750 protein-coding regions encoding 26,501,327 non-identical protein sequences, among them 25,021,987 marked as complete.

Protein clusters are built at three levels. First, tight protein clusters (80 % similarity with 85 % coverage) are built in large clades containing 10 or more non-clonal genomes using a combined approach that takes into account both sequence similarity and local genome context, and representative proteins (called *clustroids*) are selected in in-clade clusters. Then clustroids of conservative in-clade clusters are organized into medium-size (50 % similarity with 70 % coverage) *seed* global clusters, and global protein clusters are built around the *seed* clusters. The details of the algorithms are described in *Methods*.

In-clade clusters were built in 131 abundant clades containing 10 or more non-clonal assemblies. The results are summarized in Table 2.

**Table 2:** Summary of in-clade clustering for abundant clades

No. abundant clades	131
No. protein coding regions encoding complete	
proteins	19,740,968
No. non-identical protein sequences	7,604,425
No. clustroids	1,566,371
No. clustroids of conservative in-clade clusters	351,881
No. protein coding regions encoding complete	
proteins represented by clustroids	
of conservative in-clade clusters	14,612,418
No. seed global clusters	144, 415

As a result of *seed* global clustering, 144,415 *seed* clusters have been produced. They represent complete proteins encoded by 14,612,418 protein coding regions - 67 % in-clade coding regions. With the *seed* clusters we observe a substantial 10-fold level of data compression (with even higher level of compression in the largest clades).

The remaining proteins come either from non-conservative (unique) or rapidly evolving proteins, or from rare genomes. The input dataset for extended global clustering contains 19,473,537 non-identical protein sequences: 351,881 sequences are clustroids of conservative protein clusters and the rest contains clustroids of non-conservative in-clade clusters and sequences coming from the outside of the large clades. Straightforward global clustering by the modified hierarchical clustering algorithm required calculating pairwise 19,473,537×19,473,537 BLAST hits and produced 5,595,941 global clusters (where only 2.5 % of them are extended *seed* clusters, while the most of the remaining 97.5 % are low-informative groups).

Since the critical factor in processing is the calculation of BLAST hits, we first looked for ways to further decrease the number of sequences to be processed by selecting representatives in tight groups of sequences using UCLUST [25] (tight UCLUST parameters approximately correspond to the parameters used in in-clade clustering, see *Methods*). As a result, 1,263,175 protein sequences were directly assigned the *clustroids* in the *seed clusters*, while remaining 17,858,401 sequences were grouped by UCLUST in tight groups allowing selection of 11,185,110 representatives. The described reduction allows to decrease the BLAST hit calculation from 19,473,537×19,473,537 to 11,536,991×11,536,991.

The effectiveness of processing can be tremendously increased, and the amount of work dramatically reduced, if we limit ourselves to extending the *seed clusters*. In this case, we could use an approximate procedure when non-seed proteins are added to the nearest *seed* cluster if they are compatible with *seed* clustroids there. Since non-seed proteins are compared only to *seed* proteins (and are not compared to each other) in the extension procedure, only BLAST hits of 11,185,110 representatives to 351,881 *seed* sequences need to be computed. Finally, the extension procedure could be accelerated by the following filtering. UCLUST search procedure with very liberal parameters (see *Methods*) is used to find a subset of 11,185,110 proteins containing distant neighbors of the *seeds*. This subset contains 4,174,038 proteins. When we compared this subset to the elements of extended clusters, we found that 99.5 % were assigned, with a loss rate of 0.5 %. As a result, we need to calculate BLAST hits of only 4,174,038 representatives to 351,881 *seed* sequences, providing 2-fold additional acceleration in comparison to the extension procedure without filtering.

By using 50 % similarity with 70 % coverage, we considered well-established medium-size global clusters that could be further aggregated or neighbor relationships between them could be established (indeed, decrease of the minimal similarity parameter from 50 to 30 % to consider the number of *seed* clusters decreases from 144,415 *seed* clusters to 77,532 (larger) *seed* clusters).

## Discussion

We proposed a method to reduce redundancy in the 40 million prokaryotic proteins in the NCBI Microbial Genomes database. Protein clusters were created at the level of clades (organisms grouped by similarity at the species level) and the most conserved clusters were merged between the clades. Highly conserved proteins, for example those involved in cellular machinery, are conserved across taxa. Other proteins are highly conserved within well-studied large clades, for example human pathogens with extensive sequence data. This method has allowed a substantial reduction in redundancy within the microbial protein database.

The developed multilevel approach utilizing the in-clade clustering procedure, subsequent selection of clustroids, and organizing them into *seed* global clusters provides a robust representation and high rate of compression. *Seed* protein clusters are efficiently extended by adding related proteins. Extended *seed* clusters include a significant part of the data and represent all major known cell machinery. Medium-size extended *seed* clusters could be either organized in wider clusters (super-clusters) or linked together if they are related.

The remaining part of the protein dataset, known in the network theory as network periphery, comes from either non-conservative (unique) or rapidly evolving proteins, or from rare genomes, or resulting from low-quality annotations, requires significant computational resources to be processed in the clustering procedure, and results in a large number of questionable clusters. We propose filtering strategies limiting the protein dataset included in global clustering. The excluded proteins can be related as neighbors to the core clustering data through the links.

## Conclusion

The proposed method allows the analysis the relevant data at different levels of details and eliminating data redundancy while keeping biologically interesting variations.

## Additional file

Additional file 1
**Table S1.** Shows per-clade statistics for 131 abundant clades; number of proteins represents non-redundant set of non-identical protein sequences. (PDF 38 kb)
