# Supplementary material for: Clustering analysis of proteins from microbial genomes at multiple levels of resolution
Source: BMC Bioinformatics. 2016 Aug 31;17(Suppl 8):276. doi: 10.1186/s12859-016-1112-8 (PMC5009818; doi:10.1186/s12859-016-1112-8)
Supplement: Additional file 1 — Table S1. Shows per-clade statistics for 131 abundant clades; number of proteins represents non-redundant set of non-identical protein sequences. (PDF 38 kb) [file 12859_2016_1112_MOESM1_ESM.pdf]

Table S1 Per-clade statistics for 131 abundant clades; number of proteins represents non-redundant set of non-identical protein sequences

Clade Id	Taxonomic content	No. annotated genomes	No. nonclonal annotated genomes	No. protein coding regions	No. protein sequences	No. conservative inclade clusters
19668	Escherichia, Shigella	2277	929	3303114	310023	3894
19507	Acinetobacter	749	280	774670	133653	3034
19252	Helicobacter pylori	309	216	254806	191419	1244
20139	Enterococcus genus	242	155	306721	33249	2106
20104	Streptococcus genus	347	139	163066	61589	1394
20137	Enterococcus genus	300	139	309061	45809	2314
19669	Salmonella, Citrobacter	638	134	478093	112833	3940
19672	Enterobacter, Escherichia, Klebsiella	350	132	593750	84168	4726
19537	Pseudomonas	229	118	622138	100992	5511
21194	Vibrio	271	118	433416	150390	4015
19400	Neisseria genus	204	109	162808	29688	1596
19988	Staphylococcus aureus	3827	108	235562	43260	2309
20122	Streptococcus agalactiae	285	103	165898	17943	1704
19671	Enterobacter Lelliottia	80	70	229896	102783	3476
20021	Bacillus	101	70	250224	101171	3919
20103	Streptococcus suis	92	69	97200	48055	1541
19543	Pseudomonas	108	68	219354	114229	3551
19270	Campylobacter jejuni	97	63	85618	29112	1444
20116	Streptococcus mutans	165	62	100740	28671	1672
19993	Staphylococcus genus	92	59	114655	23197	2014
22045	Bacillus	72	57	204004	91370	3981
20571	Bacteroides genus	85	56	167823	43178	3185
19639	Vibrio genus	213	53	150864	58764	3075
21655	Pseudomonas	48	47	122624	96684	2993
19182	Prochlorococcus genus	47	45	44826	17488	1395
19542	Acinetobacter, Pseudomonas	46	45	138121	82184	3542
19673	Enterobacter, Escherichia, Klebsiella, Raoultella	55	45	146076	56173	3531
23492	Campylobacter coli	51	44	61804	14001	1476
20999	Leptospira interrogans	179	43	131118	31445	3318
20126	Streptococcus pyogenes	215	42	57716	18901	1440
20175	Peptoclostridium difficile	180	42	133154	31718	3307
20096	Lactobacillus casei group	52	41	83601	21892	2282
22620	Mesorhizobium genus	46	40	175832	81221	4898
19460	Burkholderia	73	38	133070	40860	4026
20017	Listeria genus	270	35	81704	32042	2559
20877	Salinispora arenicola	43	35	140603	49658	4145
19546	Pseudomonas	35	32	84606	66824	2848
19908	Ensifer, Sinorhizobium	33	32	140617	48789	4907
20774	Gardnerella vaginalis	35	32	16826	9731	613
20876	Salinispora pacifica	37	29	97783	57875	3618
19710	Haemophilus genus	30	28	34703	23136	1377
20574	Bacteroides genus	30	26	54310	30107	2364
20777	Bifidobacterium genus	29	26	35205	14218	1446
19619	Aeromonas genus	25	25	70424	48869	3272
20131	Lactococcus lactis	26	24	33477	14869	1537
19624	Vibrio genus	26	23	75313	42608	3499
20898	Streptomyces	36	23	81586	53296	3906
21132	Rhizobium genus	23	23	116850	79796	5377
19440	Bordetella genus	84	22	86637	28395	4391
19512	Acinetobacter	26	22	55878	41612	2671
20019	Bacillus cereus	25	22	91444	44235	4400
20836	Mycobacterium	71	22	89086	32179	4227
21001	Leptospira genus	23	22	62885	21383	2941
19355	Legionella pneumophila	31	21	51136	26297	2557
19487	Xanthomonas	85	21	64213	34578	3342
19660	Pantoea genus	21	21	47932	25806	2513
19678	Cronobacter genus	25	21	54225	30474	3026
20087	Lactobacillus genus	21	21	46388	18311	2451
20112	Streptococcus genus	23	21	38420	24914	1899
20200	Clostridium genus	33	21	48766	28129	2714
21018	Borrelia burgdorferi group	29	20	17156	7078	919
22103	Bacillus	33	20	64650	34772	3353
35018	Vibrio	22	20	65752	32410	3469
19463	Burkholderia	23	19	67009	55341	3901
19599	Pseudoalteromonas	21	19	43596	30425	2564
20045	Oenococcus oeni	56	19	26363	7950	1484
20580	Bacteroides, Porphyromonas	21	19	44654	18582	2633
20114	Streptococcus genus	46	18	23252	9315	1438
19485	Stenotrophomonas	16	16	48324	37422	3211
19970	Paenibacillus genus	14	16	51872	39434	3482
20042	Bacillus	28	16	52570	37376	3468
20794	Corynebacterium diphtheriae	17	16	28326	14360	1841
21015	Borrelia burgdorferi group	21	16	3414	1691	245
19214	Microcystis genus	15	15	36177	28475	2552
19920	Mesorhizobium genus	15	15	56891	48692	4069
20026	Geobacillus	17	15	35426	20704	2569
20118	Streptococcus genus	14	15	15101	9871	1060
22238	Streptomyces genus	32	15	76934	41168	5741
19709	Aggregatibacter genus	24	14	14803	10064	1185
20057	Lactobacillus genus	16	14	12895	5583	986
20097	Lactobacillus	23	14	27056	12972	2127
21126	Citrobacter, Escherichia	17	14	51376	27346	3785
22164	Rhodococcus genus	15	14	48846	32668	3771
19545	Pseudomonas	13	13	39524	28426	3282
19684	Yersinia	126	13	41072	9810	3334
20120	Streptococcus genus	14	13	16770	12555	1406
20231	Veillonella genus	13	13	14146	10428	1205
21108	Yersinia genus	24	13	34994	27761	2871
22137	Xanthomonas genus	19	13	27618	19417	2307
35123	Fusobacterium genus	13	13	19379	13100	1591
19472	Ralstonia solanacearum	12	12	34257	24562	3133
19631	Vibrio	14	12	43364	28967	3827
19697	Actinobacillus genus	19	12	19103	11804	1696
20059	Lactobacillus genus	13	12	13251	7993	1172
20185	Clostridium perfringens	12	12	28189	16443	2479
20301	Clostridium, Lachnoclostridium	17	12	32578	14075	2846
20374	Sulfolobus islandicus	20	12	25395	10208	2197
20491	Fusobacterium genus	14	12	18805	11903	1646
20568	Porphyromonas genus	13	12	16736	10492	1439
21634	Thioalkalivibrio genus	13	12	26900	16976	2296
21993	unclassified Cloacimonetes	12	12	5911	814	643
22787	Streptomyces genus	14	12	28777	26452	2532
23775	Leptospira genus	25	12	35794	15445	3053
34963	Prochlorococcus genus	12	12	13566	6026	1327
19500	Acinetobacter genus	14	11	25318	15564	2403
19587	Alteromonas genus	13	11	25976	16327	2465
19601	Colwellia, Pseudoalteromonas	11	11	26017	16837	2679
19706	Pasteurella genus	21	11	18246	9952	1815
20066	Lactobacillus genus	10	11	12290	6465	1193
20068	Lactobacillus delbrueckii	11	11	12198	8403	1182
20136	Enterococcus genus	12	11	21162	13391	2288
20829	Mycobacterium tuberculosis	1737	11	36314	13409	3536
20861	Rhodococcus genus	12	11	51809	30774	5119
20904	Streptomyces	12	11	55332	34925	5306
21006	Leptospira genus	12	11	26923	15137	2629
21637	Thioalkalivibrio genus	14	11	24747	15193	2322
21976	Acinetobacter genus	15	11	19021	13866	1796
21985	Acinetobacter	13	11	32027	24974	3030
35088	Streptomyces	11	11	38402	31338	3620
19471	Ralstonia	12	10	34594	20370	3664
19611	Shewanella genus	9	10	21595	16135	2257
19680	Pectobacterium genus	10	10	30432	22718	3143
20037	Bacillus genus	11	10	29666	22850	3094
20063	Lactobacillus crispatus	10	10	14026	5356	1506
20107	Streptococcus genus	10	10	14364	11618	1499
20125	Streptococcus	10	10	12091	7568	1324
20130	Lactococcus garvieae	13	10	14383	8728	1522
20831	Mycobacterium avium	32	10	28359	12951	3091
20998	Leptospira genus	10	10	26944	16321	2889
21023	Treponema denticola	17	10	19239	14000	2000
23759	Salinispora genus	11	10	39931	12983	4063
